# Effects of αβ-Blocker Versus β1-Blocker Treatment on Heart Rate Response During Incremental Cardiopulmonary Exercise in Japanese Male Patients with Subacute Myocardial Infarction

**DOI:** 10.3390/ijerph16162838

**Published:** 2019-08-08

**Authors:** Shinji Nemoto, Yusuke Kasahara, Kazuhiro P. Izawa, Satoshi Watanabe, Kazuya Yoshizawa, Naoya Takeichi, Kentaro Kamiya, Norio Suzuki, Kazuto Omiya, Atsuhiko Matsunaga, Yoshihiro J. Akashi

**Affiliations:** 1 Department of Rehabilitation Medicine, St. Marianna University School of Medicine Yokohama City Seibu Hospital, Yokohama 241-0811 Japan; 2 Department of Rehabilitation Sciences, Kitasato University Graduate School of Medical Sciences, Sagamihara 252-0373, Japan; 3 Department of Public Health, Kobe University Graduate School of Health Sciences, Kobe 654-0142, Japan; 4 Department of Rehabilitation Medicine, St. Marianna University School of Medicine Hospital, Kawasaki 216-8511, Japan; 5 Department of Rehabilitation Medicine, Kawasaki Municipal Tama Hospital, Kawasaki 214-8525, Japan; 6 Division of Cardiology, Department of Internal Medicine, St. Marianna University School of Medicine Yokohama City Seibu Hospital, Yokohama 241-0811, Japan; 7 Department of Internal Medicine, Shimazu Medical Clinic, Yokohama 226-0026, Japan; 8 Division of Cardiology, Department of Internal Medicine, St. Marianna University School of Medicine Hospital, Kawasaki 216-8511, Japan

**Keywords:** myocardial infarction, beta-blocker, metabolic chronotropic relationship, chronotropic index, heart rate response, cardiac rehabilitation

## Abstract

A simplified substitute for heart rate (HR) at the anaerobic threshold (AT), i.e., resting HR plus 30 beats per minute or a percentage of predicted maximum HR, is used as a way to determine exercise intensity without cardiopulmonary exercise testing (CPX) data. However, difficulties arise when using this method in subacute myocardial infarction (MI) patients undergoing beta-blocker therapy. This study compared the effects of αβ-blocker and β1-blocker treatment to clarify how different beta blockers affect HR response during incremental exercise. MI patients were divided into αβ-blocker (*n* = 67), β1-blocker (*n* = 17), and no-β-blocker (*n* = 47) groups. All patients underwent CPX one month after MI onset. The metabolic chronotropic relationship (MCR) was calculated as an indicator of HR response from the ratio of estimated HR to measured HR at AT (MCR-AT) and peak exercise (MCR-peak). MCR-AT and MCR-peak were significantly higher in the αβ-blocker group than in the β1-blocker group (*p* < 0.001, respectively). Multiple regression analysis revealed that β1-blocker but not αβ-blocker treatment significantly predicted lower MCR-AT and MCR-peak (β = −0.432, *p* < 0.001; β = −0.473, *p* < 0.001, respectively). Based on these results, when using the simplified method, exercise intensity should be prescribed according to the type of beta blocker used.

## 1. Introduction

Many guidelines recommend exercise training for patients with myocardial infarction (MI) [1,2] because exercise training has been associated with reduced cardiovascular mortality after acute MI [3]. When prescribing exercise for subacute MI patients, it is important to determine the optimum exercise intensity to ensure safe and effective training. The recommended technique for determining the target heart rate (HR) is by measuring HR at the anaerobic threshold (AT) via cardiopulmonary exercise testing (CPX). Exercise prescription using HR at AT is effective and safe for MI patients [4,5]. Although prescribing exercise intensity based on the AT measured by CPX is desirable, it is not easy to perform CPX for all patients due to staffing requirements and the high cost of equipment. It is recommended to first perform an exercise stress test to determine the target HR range by the Karvonen formula, if gas-exchange analysis data cannot be obtained [2]. Meanwhile, a simplified substitute for HR at AT, i.e., resting HR plus 30 beats per minute (bpm) or a percentage of predicted maximum HR (% predicted max HR), is used as a way to determine exercise intensity without gas-exchange analysis data or exercise stress test data [2,6]. This simplified method, however, is considered difficult to use if patients are undergoing beta-blocker treatment, since beta blockers reportedly affect calculations by reducing HR responses during incremental exercise [7,8]. Although the negative chronotropic effect of beta blockers is considered to differ depending on beta-blocker type (e.g., αβ-blocker or β1-blocker), previous studies have not fully evaluated the effects of beta-blocker type on HR response during incremental exercise in subacute MI patients. The effect of beta-blocker type on this simplified method of exercise prescription, therefore, remains unclear. The present study aimed to compare the effects of αβ-blockers and β1-blockers on HR response during incremental exercise in subacute MI patients, because both αβ-blockers and β1-blockers are frequently prescribed for MI patients due to their effectiveness [9,10,11]. Clarifying differences in the effects of αβ-blockers and β1-blockers on HR response would help physicians to prescribe appropriate exercise intensities for subacute MI patients who are undergoing beta-blocker treatment.

## 2. Materials and Methods

### 2.1. Study Design and Patients

A total of 665 consecutive acute MI patients underwent cardiac rehabilitation at St. Marianna University School of Medicine, Yokohama City Seibu Hospital, between June 1998 and March 2018. Of these, 297 were able to undergo symptom-limited CPX one month after the onset of MI, and 131 who did not meet the following exclusion criteria were enrolled in this retrospective cross-sectional study: the presence of atrial fibrillation, complex arrhythmia, or pacemaker; recent myocardial infarction; history of cardiac surgery; female sex; maximum respiratory exchange ratio (RER) during CPX < 1.10; and use of drugs other than beta blockers with positive or negative chronotropic effects (e.g., verapamil, diltiazem hydrochloride). Patients were divided into the following three groups according to the type of beta blocker used: the αβ-blocker group (67 patients), the β1-blocker group (17 patients), and the no-β-blocker group (47 patients). All patients were clinically stable and were examined while being treated with stable doses of medication. 

The study protocol conformed to the ethical guidelines of the Declaration of Helsinki and was approved by the Ethics Committee of St. Marianna University School of Medicine (Chairperson of the Ethics Committee, Masashi Taki; Protocol No. 2258; date of approval by the Ethics Committee, November 30, 2012). Informed consent was obtained from all patients before conducting CPX.

### 2.2. Clinical Characteristics

Age, body mass index (BMI), site of infarction, residual coronary artery stenosis, past medical history, complications, medications, beta-blocker dose, time between MI onset and exercise testing, and year of hospitalization were evaluated through a review of patient medical records. The ratio of administered beta-blocker dose to maximum beta-blocker dose (% maximum beta-blocker dose) was calculated by dividing the administered beta-blocker dose by the maximum beta-blocker dose for Japanese patients (e.g., carvedilol 20 mg/day, bisoprolol 5 mg/day, and atenolol 50 mg/day). Maximum serum creatine kinase-myocardial band (max CK-MB) was measured using blood samples obtained during hospitalization. Left ventricular ejection fraction (LVEF) was echocardiographically determined by a modified Simpson’s method and used as the index of left ventricular systolic function at the time of hospital discharge.

### 2.3. Symptom-Limited CPX

All patients underwent symptom-limited CPX using a treadmill (MAT-2700, Fukuda Denshi Co., Tokyo, Japan). After three minutes of resting in a seated position, patients performed exercises with an increasing load (speed or grade) every 60 s after three minutes of warm-up activity (1 mph speed, 0% grade [12]. A 12-lead electrocardiogram was continuously monitored, and HR was measured from the R-R interval of the electrocardiogram (ML-9000, Fukuda Denshi Co., Tokyo, Japan) during the test. Systolic blood pressure (SBP) and diastolic blood pressure (DBP) were measured using a blood pressure cuff and an automatic blood pressure monitor (STBP-780, Colin Co., Aichi, Japan) at one-minute intervals. Oxygen uptake (VO_2_), carbon dioxide production, minute ventilation, tidal volume, end-tidal O_2_, and end-tidal CO_2_ were measured throughout the test using an aeromonitor (AE-310s, Minato Ikagaku Co., Tokyo, Japan). Expired gas was sampled using a breath-by-breath method. The endpoint of testing was determined as previously described [13]. Specifically, the appearance of a plateau in VO_2_ despite an increasing exercise intensity indicated the exercise endpoint. 

Ventilatory equivalents were calculated for O_2_, CO_2_, and RER using a personal computer (Model PC-9801, NEC Co., Tokyo, Japan). AT was determined by the V-slope method [14].

HR at rest (HR_rest_), HR at AT (HR_AT_), and HR at peak VO_2_ (HR_peak_) were determined from the recorded HR during the course of CPX. Increases from HR_rest_ to HR_peak_ (Δpeak HR), from HR_rest_ to HR_AT_ (ΔAT HR), and from HR_AT_ to HR_peak_ (ΔAT-peak HR) were calculated for all patients.

### 2.4. Calculation of the Chronotropic Index

The metabolic chronotropic relationship (MCR), also referred to as the chronotropic index, was calculated as an indicator of HR response during incremental exercise at each stage (i.e., AT and peak VO_2_) based on the relationship between HR and VO_2_ during exercise [15,16,17]. The MCR, proposed by Wilkoff, allows for comparison between patients irrespective of age, physical fitness, functional capacity, exercise testing mode, or protocol. Estimated HR was calculated at AT and peak VO_2_ using the following formula:

Estimated HR_stage_ = (220 − age − HR_rest_) × [(METs_stage_ − 1) / (METs_peak_ − 1)] + HR_rest_, (1)

where metabolic equivalents (METs) were defined as VO_2_ (ml/kg/min) divided by 3.5. The chronotropic index at AT (MCR-AT) was calculated as the ratio of estimated HR_AT_ to measured HR_AT_, and at peak VO_2_ (MCR-peak) was calculated as the ratio of estimated HR_peak_ to measured HR_peak_, as follows:

MCR-AT = HR_AT_ / estimated HR_AT_, (2)

MCR-peak = HR_peak_ / estimated HR_peak_, (3)

MCR-AT and MCR-peak were used as indicators of HR response to incremental exercise up to the AT and peak exercise, respectively. A chronotropic index < 0.8 was previously determined to indicate chronotropic incompetence [15,16].

### 2.5. Statistical Analysis

Results are expressed as mean ± standard error (SE). The Kolmogorov–Smirnov test was performed to assess the normality of distribution. Differences in clinical characteristics, CPX results, and MCR findings among the three groups were analyzed using one-way analysis of variance (ANOVA), χ^2^ test, and Tukey’s honestly significant difference test. Tukey’s honestly significant difference test was used if the results of one-way ANOVA were statistically significant. Pearson’s correlation coefficient and Spearman’s rank correlation coefficient were used in a regression analysis to determine correlations between MCR-AT, MCR-peak, and clinical characteristics (age and BMI were excluded from correlations as the formula of MCR included these factors), and correlations in which *p* < 0.2 were considered statistically significant. Correlations, clinically important factors on the nominal scale, αβ-blocker treatment, and β1-blocker treatment were entered into a multiple regression model for predictions of MCR-AT or MCR-peak using the forced-entry method. For patients undergoing beta-blocker treatment, Spearman’s rank correlation coefficient was used to examine correlations between MCR-AT, MCR-peak, and % maximum beta-blocker dose. *p* < 0.05 was considered statistically significant. All statistical analyses were performed with JMP^®^ Pro 13 (SAS Institute Inc., Cary, NC, USA). A priori power analysis was performed for multiple regression analysis using G*Power 3 (Heinrich-Heine-Universität, Düsseldorf, Germany). The sample size was calculated to be 123 in the regression model for MCR-peak (effect size = 0.15; α error probability = 0.05; power (1−β error probability) = 0.8; number of predictors = 11). Accordingly, the sample size of the present study was considered sufficient for conducting multiple regression analysis. Effect sizes were also calculated using G*Power 3. 

## 3. Results

Table 1 shows the clinical characteristics of patients included in this study. Significant differences between αβ-blocker, β1-blocker, and no-β-blocker groups were observed in renin-angiotensin system inhibitor treatment and hospitalization before 2006 (*p* = 0.024 and *p* < 0.001, respectively). The value of % maximum beta-blocker dose was significantly lower in the αβ-blocker group compared to the β1-blocker group (*p* < 0.001). No significant differences were observed in other clinical characteristics between the three groups.

Table 2 summarizes the data obtained during CPX. None of the patients showed ischemic ST changes or experienced chest pain or serious arrhythmias during CPX. The AT and peak VO_2_ did not differ significantly between the three groups (*p* = 0.297 and *p* = 0.276, respectively). HR_AT_, HR_peak_, ΔAT HR, and Δpeak HR were significantly higher in the αβ-blocker group compared to the β1-blocker group (*p* = 0.004, *p* = 0.002, *p* = 0.004, and *p* = 0.003, respectively). HR_rest_, HR_AT_, and HR_peak_ were significantly lower in the αβ-blocker group compared to the no-β-blocker group (*p* < 0.001, *p* = 0.014, and *p* = 0.002, respectively). HR_rest_, HR_AT_, HR_peak_, Δpeak HR, and ΔAT-peak HR were significantly lower in the β1-blocker group compared to the no-β-blocker group (*p* < 0.001, *p* < 0.001, *p* < 0.001, *p* = 0.001, and *p* = 0.011, respectively). MCR-AT and MCR-peak were both significantly higher in the αβ-blocker group compared to the β1-blocker group (*p* < 0.001, effect size (d) = 1.143; *p* < 0.001, d = 1.215, respectively). However, compared to the no-β-blocker group, MCR-peak was significantly lower in the αβ-blocker group (*p* = 0.038, d = 0.448), although no significant difference was observed in MCR-AT between the two groups (*p* = 0.406, d = 0.279). Both MCR-AT and MCR-peak were significantly lower in the β1-blocker group compared to the no-β-blocker group (*p* < 0.001, d = 1.429; *p* < 0.001, d = 1.346, respectively). 

The results of the regression analysis revealed no correlation between MCR-AT and Log max CK-MB, LVEF, and time between MI onset and exercise testing. MCR-peak was positively correlated with Log max CK-MB (*p* = 0.188), and negatively correlated with LVEF (*p* = 0.053). Multiple regression analysis revealed that β1-blocker but not αβ-blocker treatment significantly predicted lower MCR-AT and MCR-peak (β = −0.432, *p* < 0.001; β = −0.473, *p* < 0.001, respectively) (Table 3 and Table 4).

**Figure 1:**
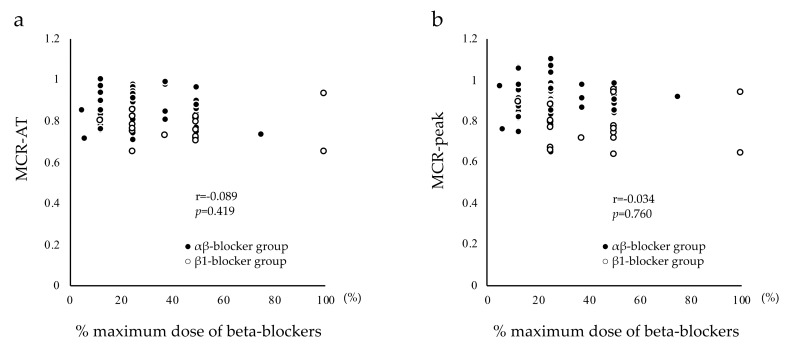
Correlations between MCR-AT, MCR-peak, and % maximum dose of beta blockers. Figure 1a shows correlation results between MCR-AT and % maximum beta-blocker dose. In patients undergoing beta-blocker treatment, no significant correlations were observed between MCR-AT and % maximum beta-blocker dose. Figure 1b shows correlation results between MCR-peak and % maximum beta-blocker dose. In patients undergoing beta-blocker treatment, no significant correlations were observed between MCR-peak and % maximum beta-blocker dose. MCR, metabolic chronotropic relationship; MCR-AT, ratio of estimated heart rate at the AT to measured heart rate at the AT; MCR-peak, ratio of estimated heart rate at peak oxygen uptake to measured heart rate at peak oxygen uptake; % maximum beta-blocker dose, ratio of administered beta-blocker dose to maximum beta-blocker dose.

Figure 1a and 1b show the correlation results between MCR-AT, MCR-peak, and % maximum beta-blocker dose. In patients undergoing beta-blocker treatment, no significant correlations were observed between MCR-AT, MCR-peak, and % maximum beta-blocker dose (*p* = 0.419, r = −0.089; and *p* = 0.760, r = −0.034, respectively).

## 4. Discussion

The present study compared the effects of β1-blocker versus αβ-blocker treatment on HR response during incremental CPX in subacute MI patients, using MCR as an indicator of HR response. Both MCR-AT and MCR-peak were significantly lower in the β1-blocker group than in the αβ-blocker group. Multiple regression analysis revealed that β1-blocker but not αβ-blocker treatment significantly predicted lower MCR-AT and MCR-peak. These results suggest that the negative chronotropic effect of beta blockers during incremental CPX may be stronger with β1-blockers than with αβ-blockers in subacute MI patients.

### 4.1. Selecting Beta-Blocker Type for Subacute MI Patients

Existing guidelines recommend administration of beta blockers to MI patients in order to reduce post-MI mortality [2,18]. However, there are no criteria for selecting a specific type of beta blocker for MI patients. A previous study reported no differences in the risk of all-cause death or MI between MI patients treated with αβ-blockers versus β1-blockers [10]. In the present study, the type of beta blocker used (i.e., αβ-blocker or β1-blocker) was determined based on clinical decisions made by physicians. Despite guideline recommendations, some patients were not treated with beta blockers. This is likely because, in Japan, the administration of beta blockers was first recommended by the Guidelines for Secondary Prevention of Myocardial Infarction in 2006 [19]. In fact, most patients who did not undergo beta-blocker treatment were hospitalized prior to 2006.

### 4.2. Differences in HR Response During CPX Among Subacute MI Patients Undergoing αβ-Blocker Versus β1-Blocker Treatment

HR responses during incremental exercise are greatly affected by cardiac autonomic nerve activity. When exercise intensity is low to moderate, HR response is considered to be mainly affected by decreased parasympathetic nervous system activity; when exercise intensity is moderate to high, HR response is considered to be mainly affected by increased sympathetic nervous system activity [20]. Increases in HR from rest to maximal exercise result from a transition in workload from a 4:1 parasympathetic / sympathetic balance to a 1:4 parasympathetic / sympathetic balance [20]. Among MI patients, the autonomic nerve balance is considered to trend more toward increasing sympathetic nervous system activity due to unbalanced cardiac autonomic modulation (i.e., increased sympathetic outflow and reduced parasympathetic activity) [21]. Beta blockers decrease sympathetic nervous system activity by inhibiting the interactions between noradrenaline and beta receptors. β1-blockers selectively inhibit the interaction between noradrenaline and β1 receptors in the heart, while αβ-blockers non-selectively inhibit the interactions between noradrenaline and β1 receptors. The effect of αβ-blockers to decrease cardiac sympathetic nervous system activity is thus considered to be lower than that of β1-blockers. In the present study, MCR-AT and MCR-peak were significantly lower in the αβ-blocker group than in the β1-blocker group. A chronotropic index < 0.8 has been previously determined to indicate chronotropic incompetence [15,16]. In the present study, the values of MCR-AT and MCR-peak were 0.85 ± 0.07 and 0.90 ± 0.08, respectively, in the αβ-blocker group, and 0.77 ± 0.07 and 0.78 ± 0.11, respectively, in the β1-blocker group. These results show that the β1-blocker group had a low chronotropic index, while the αβ-blocker group did not, suggesting that the effect of β1-blockers decreasing HR response during incremental CPX may be stronger than with αβ-blockers. 

### 4.3. Effects of αβ-Blocker Versus β1-Blocker Treatment on HR Response During CPX in Subacute MI Patients After Adjusting for Multiple Factors

Previous studies suggested that several factors other than beta-blocker treatment could affect HR response during exercise, including age, body weight, functional capacity, cardiac function, high max CK-MB, diabetes mellitus, and heart failure after MI [22,23,24,25,26]. The present study used MCR as an indicator of HR response, because MCR allows for the assessment of HR response without the effects of age, physical fitness, or functional capacity [15,16]. In order to adjust for other several factors, multiple regression analysis was performed to predict MCR. The analysis revealed that β1-blocker but not αβ-blocker treatment significantly predicted lower MCR-AT and MCR-peak.

### 4.4. Beta-Blocker Doses

In the present study, the mean dose of carvedilol was 5.48 ± 2.99 mg/day in the αβ-blocker group, and mean doses of bisoprolol and atenolol were 2.50 ± 1.40 and 33.33 ± 12.91 mg/day, respectively, in the β1-blocker group. A previous study reported that Japanese MI patients were treated with a mean carvedilol dose of 3.48 ± 1.61 mg/day before hospital discharge and 7.71 ± 4.58 mg/day at 12 months after MI onset, or a mean bisoprolol dose of 1.26 ± 0.57 mg/day before hospital discharge and 2.47 ± 1.20 mg/day at 12 months after MI onset [11]. Another study in Japan reported that atenolol was started at a low dose and titrated up to the target dose of 25–50 mg/day at one month after MI onset [27]. In the present study population (i.e., MI patients at one month after MI onset), although the mean doses of carvedilol and atenolol were considered clinically appropriate, the mean dose of bisoprolol was slightly higher than that noted in previous study populations. To examine the effect of beta-blocker doses on HR response, we also examined the correlations between MCR-AT, MCR-peak, and % maximum beta-blocker dose. No significant correlations were observed between MCR-AT, MCR-peak, and % maximum beta-blocker dose. The negative chronotropic effect of beta blockers depends on the concentration of these medications in the blood. In addition to dose, several clinical factors can affect the blood concentration of beta blockers, such as body weight, renal function, liver function, and genetic polymorphism [28,29,30]. In clinical practice, physicians determine appropriate doses for each MI patient based on these factors. In other words, optimum beta-blocker doses for blood concentration are different for each patient. Thus, no significant correlations were observed between MCR-AT, MCR-peak, and % maximum beta-blocker dose in the present study. However, from the results of the present study, we could not conclude that there is no relationship between the dose of a β-blocker and HR response. Because it is difficult to assess whether each patient was on a low, moderate, or high dose relative to the individual response to treatment, the effect of beta-blockers on each patient was not assessed in the present study.

### 4.5. Clinical Application of Study Findings

In clinical practice, a simplified method of determining exercise intensity without gas-exchange analysis data or exercise stress test data is to use a substitute for HR at AT, i.e., resting HR plus 30 bpm or % predicted max HR. [2,6]. This simplified method is considered difficult to use in patients undergoing beta-blocker treatment, based on reports from previous studies that beta blockers affect calculations by reducing HR responses during incremental exercise [7,8]. Although the negative chronotropic effect of beta blockers is considered to differ depending on beta-blocker type (e.g., αβ-blocker or β1-blocker), previous studies have not fully evaluated the effects of beta-blocker type on HR response during incremental exercise in subacute MI patients. The effect of beta-blocker type on this simplified method of exercise prescription, therefore, remains unclear. Our previous study [31] examined the effect of αβ-blocker treatment on HR response during incremental exercise in subacute MI patients, because αβ-blockers are frequently prescribed for MI patients due to their effectiveness [9]. αβ-blocker treatment was found to have no effect on HR response to exercise up to the AT in subacute MI patients [31], suggesting that exercise intensity can be prescribed using the simplified method (i.e., resting HR plus 30 bpm) in subacute MI patients regardless of αβ-blocker use. Although β1-blockers are also frequently used in MI patients [10,11], the effect of β1-blockers on HR response during incremental exercise in subacute MI patients remains unclear. The present study compared the effects of β1-blocker versus αβ-blocker treatment on HR response during incremental exercise in subacute MI patients, and found that both MCR-AT and MCR-peak values were significantly lower in the β1-blocker group than in the αβ-blocker group. These results suggest that, when prescribing exercise intensity for subacute MI patients in clinical practice based on calculations of resting HR plus 30 bpm or % predicted max HR, we may need to use a lower target HR/predicted max HR value for those who are undergoing β1-blocker treatment, compared to those who are undergoing αβ-blocker treatment. In order to determine an appropriate target HR value for subacute MI patients undergoing β1-blocker treatment, further evaluation of training effectiveness and safety is needed. A previous study presented a prediction formula for maximum HR among patients with coronary heart disease receiving beta-blocker treatment (i.e., predicted maximum HR = 164 − 0.7 × age) [6]. However, this prediction formula was not specific for each type of beta blocker. Prediction formulas for maximum HR in MI patients treated with each type of beta blocker need to be established in future studies.

### 4.6. Limitations

The present study has several limitations. First, the influence of potential factors (e.g., body weight, renal function, liver function, genetic polymorphism) on the blood concentration of beta blockers was not examined in the present study. Second, all patients underwent symptom-limited CPX using a treadmill. The effects of beta-blocker type on HR response during other symptom-limited CPX (e.g., on a bicycle ergometer) have not yet been clarified. Third, this study was analyzed retrospectively. Hence, the effect of several biases such as an imbalance in the number of patients and a difference in the treatment of MI between the groups cannot be completely excluded in this study. A future randomized controlled trial will be needed to verify the results of the present study. Finally, this study is affected by selection bias as patients who were unable to undergo symptom-limited CPX on a treadmill due to frailty were excluded from the study. As a result, the population in this study comprised lean and young patients. A future study will be needed to verify the results of the present study in a broader age range of patients.

## 5. Conclusions

Our data suggest that the effects of β1-blockers to decrease HR response during incremental CPX may be stronger than that of αβ-blockers in subacute MI patients. Therefore, exercise intensity based on a simplified substitute for HR at AT (i.e., resting HR plus 30 bpm or % predicted max HR) without gas-exchange analysis data or exercise stress test data should be prescribed according to the type of beta blocker used in subacute MI patients.

## Figures and Tables

**Table 1:** Patient clinical characteristics.

Clinical Characteristics	αβ-Blocker Group (*n* = 67)	β1-Blocker Group (*n* = 17)	No-β-Blocker Group (*n* = 47)	*p*-Value
Age (years)	64.98 ± 10.48	61.59 ± 8.91	61.62 ± 10.83	0.184
Body mass index (kg/m^2^)	23.44 ± 2.78	23.58 ± 3.27	23.12 ± 3.10	0.804
MI				0.621
Inferior	23 (37.3)	7 (41.2)	24 (51.1)	
Anterior	34 (50.8)	9 (52.9)	19 (40.4)	
Lateral	8 (11.9)	1 (5.9)	4 (8.5)	
Residual coronary artery stenosis	30 (44.9)	11 (64.7)	19 (40.4)	0.221
Medical history				
Prior MI	4 (5.8)	2 (11.8)	4 (8.5)	0.696
Hypertension	48 (71.6)	13 (76.5)	31 (66.0)	0.673
Dyslipidemia	39 (58.2)	12 (70.6)	30 (63.8)	0.605
Chronic kidney disease	7 (10.5)	2 (11.8)	6 (12.8)	0.929
Diabetes mellitus	19 (28.4)	4 (23.5)	19 (40.4)	0.287
Orthopedic disorder	2 (3.0)	1 (6.3)	0 (0)	0.309
Cerebrovascular disease	1 (1.5)	0 (0)	3 (6.4)	0.241
Respiratory disease	2 (3.0)	0 (0)	1 (2.1)	0.760
Hyperuricemia	7 (10.5)	3 (17.7)	8 (17.0)	0.533
Peripheral arterial disease	1 (1.5)	1 (5.9)	1 (2.1)	0.555
Dementia	0 (0)	0 (0)	1 (2.1)	0.406
Heart failure after MI	6 (9.0)	1 (5.9)	5 (10.6)	0.841
Medication				
Diuretic	9 (13.4)	2 (11.8)	4 (8.5)	0.718
Renin-angiotensin system inhibitor	63 (94.0)	12 (70.6)	41 (87.2)	0.024
Calcium antagonist	12 (17.9)	1 (5.9)	3 (6.4)	0.125
Aldosterone antagonist	5 (7.5)	3 (17.6)	2 (4.3)	0.204
Anticlotting drug	2 (3.0)	1 (5.9)	1 (2.1)	0.742
Antiplatelet drug	67 (100)	17 (100)	47 (100)	1.00
αβ-blocker, Carvedilol	67 (100)	-	-	-
β1-blocker, Bisoprolol/Atenolol	-	11 (64.7)/6 (35.3)	-	-
Beta-blocker dose				
Carvedilol (mg/day)	5.48 ± 2.99	-	-	-
Bisoprolol/Atenolol (mg/day)	-	2.50 ± 1.40/33.33 ± 12.91	-	-
% maximum dose of beta-blocker (%)	27.41 ± 14.94	44.12 ± 24.65	-	<0.001
Max CK-MB (ng/ml)	306.70 ± 228.05	232.01 ± 214.07	275.43 ± 225.89	0.477
Log max CK-MB	5.40 ± 0.90	5.03 ± 1.08	5.18 ± 0.10	0.262
LVEF (%)	52.91 ± 8.81	57.94 ± 13.60	54.34 ± 12.28	0.230
Time between MI and CPX (days)	32.09 ± 11.50	27.76 ± 8.72	31.19 ± 12.35	0.387
Hospitalization before 2006	7 (10.4%)	10 (58.8%)	40 (85.1%)	<0.001

Table 1 shows comparison results for patient clinical characteristics in the αβ-blocker group, the β1-blocker group, and the no-β-blocker group. Values are expressed as mean ± standard deviation (SD), number (%), or number / number. CPX, cardiopulmonary exercise testing; LVEF, left ventricular ejection fraction; Max CK-MB, maximum value of serum creatine kinase-myocardial band; MI, myocardial infarction.

**Table 2:** Cardiopulmonary exercise testing data with gas analysis.

Cardiopulmonary Exercise Testing Data	β-Blocker Group (*n* = 67)	β1-Blocker Group (*n* = 17)	No-β-Blocker Group (*n* = 47)	F-Value	*p*-Value
Average time of exercise (min)	7.03 ± 1.57	7.27 ± 1.63	7.53 ± 1.61	1.372	0.402
AT (ml/kg/min)	15.92 ± 3.24	14.72 ± 2.24	15.91 ± 2.73	1.226	0.297
Peak VO_2_ (ml/kg/min)	23.27 ± 5.32	21.47 ± 3.72	23.66 ± 4.42	1.302	0.276
RER_AT_	0.89 ± 0.05	0.89 ± 0.05	0.89 ± 0.07	0.033	0.968
RER_peak_	1.20 ± 0.07	1.20 ± 0.07	1.18 ± 0.06	2.086	0.128
HR_rest_ (bpm)	71.49 ± 9.86 ^††^	66.65 ± 11.14 ^††^	79.02 ± 11.39	11.147	<0.001
HR_AT_ (bpm)	105.76 ± 11.19 ^‡‡^	95.47 ± 10.20 ^††^	111.19 ± 12.24 ^§^	11.940	<0.001
HR_peak_ (bpm)	138.76 ± 14.59 ^‡‡^	123.77 ± 18.80 ^††^	148.38 ± 16.21 ^§§^	15.865	<0.001
SBP_rest_ (mmHg)	127.43 ± 16.14 ^†^	129.77 ± 18.91	119.64 ± 17.23	3.734	0.027
SBP_AT_ (mmHg)	158.49 ± 21.23	151.77 ± 27.66	149.30 ± 29.78	1.904	0.153
SBP_peak_ (mmHg)	185.93 ± 28.23	180.00 ± 29.19	183.40 ± 32.46	0.295	0.745
DBP_rest_ (mmHg)	77.03 ± 12.16	84.29 ± 10.73 ^††^	74.02 ± 10.39	5.099	0.007
DBP_AT_ (mmHg)	76.00 ± 17.21	80.24 ± 10.13	76.81 ± 11.83	0.575	0.564
DBP_peak_ (mmHg)	84.51 ± 18.20	85.65 ± 15.96	82.77 ± 12.83	0.2576	0.773
ΔAT HR (bpm)	34.27 ± 8.79 ^‡^	28.82 ± 8.10	32.17 ± 7.76	3.098	0.049
Δpeak HR (bpm)	67.27 ± 13.43 ^‡^	57.12 ± 13.97 ^†^	69.36 ± 16.44	4.464	0.013
ΔAT-peak HR (bpm)	32.76 ± 9.99	28.29 ± 11.46 ^†^	37.41 ± 12.11	4.965	0.008
MCR-AT	0.85 ± 0.07 ^‡‡^	0.77 ± 0.07 ^††^	0.87 ± 0.07	13.122	<0.001
MCR-peak	0.90 ± 0.08 ^‡‡^	0.78 ± 0.11 ^††^	0.94 ± 0.09 ^§^	19.205	<0.001

Comparison results of variables during cardiopulmonary exercise testing with gas analysis in the αβ-blocker group, the β1-blocker group, and the no-β-blocker group were analyzed using one-way analysis of variance and Tukey’s honestly significant difference test. Values are expressed as mean ± standard deviation (SD). AT, anaerobic threshold; DBP, diastolic blood pressure; DBP_AT_, DBP at AT; DBP_peak_, DBP at peak oxygen uptake; DBP_rest_, DBP at rest; RER, Respiratory exchange ratio; RER_AT_, RER at AT; RER_peak_, RER at peak oxygen uptake; HR, heart rate; HR_AT_, HR at AT; HR_peak_, HR at peak oxygen uptake; HR_rest_, HR at rest; MCR, metabolic chronotropic relationship; MCR-AT, estimated heart rate at AT / measured heart rate at AT; MCR-peak, estimated heart rate at peak / measured heart rate at peak; Peak VO_2_, peak oxygen uptake; SBP, systolic blood pressure; SBP_AT_, SBP at AT; SBP_peak_, SBP at peak VO_2_; SBP_rest_, SBP at rest; ΔAT HR, (HR_AT_) − (HR_rest_); Δpeak HR, (HR_peak_) − (HR_rest_); ΔAT-peak HR, (HR_peak_) − (HR_AT_); ^†^, *p* < 0.05 vs. no-β-blocker group; ^††^, *p* < 0.01 vs. no-β-blocker group; ^‡^, *p* < 0.05 vs. β1-blocker group; ^‡‡^, *p* < 0.01 vs. β1-blocker group; ^§^, *p* < 0.05 vs. αβ-blocker group; ^§§^, *p* < 0.01 vs. αβ-blocker group.

**Table 3:** Results of multiple regression analysis for the prediction of MCR-AT.

Independent Variables	Dependent Variable: MCR-AT			
	B ± SE	β	95% CI of B	*p*-Value
Inferior infarct *	−0.002 ± 0.012	−0.025	−0.025 to 0.021	0.865
Anterior infarct *	−0.001 ± 0.011	−0.001	−0.023 to 0.022	0.946
Residual coronary artery stenosis	−0.009 ± 0.007	−0.117	−0.022 to 0.004	0.170
Heart failure after MI	−0.007 ± 0.011	−0.053	−0.029 to 0.015	0.518
Diabetes mellitus	0.001 ± 0.007	0.016	−0.012 to 0.015	0.836
Renin-angiotensin system inhibitor	−0.004 ± 0.010	−0.031	−0.025 to 0.017	0.719
αβ-blocker treatment **	−0.008 ± 0.010	−0.108	−0.028 to 0.011	0.394
β1-blocker treatment **	−0.050 ± 0.011	−0.432	−0.071 to −0.028	<0.001
Hospitalization before 2006	0.000 ± 0.001	0.003	−0.018 to 0.018	0.979
Constant	0.807 ± 0.016	0.000	0.776 to 0.839	<0.001
	Coefficient of determination R^2^ = 0.187, F = 3.092, *p* = 0.002			

Multiple regression analysis for the prediction of MCR-AT was performed using the forced-entry method. MCR, metabolic chronotropic relationship; AT, anaerobic threshold; MCR-AT, measured heart rate at AT / estimated heart rate at AT; B ± SE, partial regression coefficient ± standard error; β, standardized partial regression coefficient; CI, confidence interval; MI, myocardial infarction. * Compared with lateral infarct. ** Compared with not undergoing beta-blocker treatment.

**Table 4:** Results of multiple regression analysis for the prediction of MCR-peak.

Independent Variables	Dependent Variable: MCR-Peak			
	B ± SE	β	95% CI of B	*p*-Value
Inferior infarct *	−0.002 ± 0.015	−0.017	−0.031 to 0.027	0.907
Anterior infarct *	−0.001 ± 0.001	−0.007	−0.029 to 0.028	0.962
Residual coronary artery stenosis	−0.012 ± 0.008	−0.114	−0.028 to 0.005	0.166
Heart failure after MI	0.002 ± 0.015	0.012	−0.026 to 0.031	0.883
Diabetes mellitus	0.004 ± 0.009	0.038	−0.013 to 0.021	0.642
Log max CK-MB	0.018 ± 0.024	0.067	−0.029 to 0.064	0.449
LVEF	−0.027 ± 0.025	−0.100	−0.077 to 0.021	0.266
Renin-angiotensin system inhibitor	0.003 ± 0.013	0.021	−0.023 to 0.030	0.800
αβ-blocker treatment **	−0.019 ± 0.012	−0.185	−0.043 to 0.006	0.134
β1-blocker treatment **	−0.071 ± 0.014	−0.473	−0.099 to −0.044	<0.001
Hospitalization before 2006	−0.004 ± 0.012	−0.043	−0.019 to 0.027	0.706
Constant	0.844 ± 0.020	0.000	0.840 to 0.884	<0.001
	Coefficient of determination R^2^ = 0.263, F = 3.865, *p* < 0.001			

Multiple regression analysis for the prediction of MCR-peak was performed using the forced-entry method. MCR, metabolic chronotropic relationship; MCR-peak, measured heart rate at peak/estimated heart rate at peak; B ± SE, partial regression coefficient ± standard error; β, standardized partial regression coefficient; CI, confidence interval; LVEF, left ventricular ejection fraction; Max CK-MB, maximum value of serum creatine kinase-myocardial band; MI, myocardial infarction. * Compared with lateral infarct. ** Compared with not receiving beta-blocker treatment.
